# Co-Designing a Web-Based and Tablet App to Evaluate Clinical Outcomes of Early Psychosis Service Users in a Learning Health Care Network: User-Centered Design Workshop and Pilot Study

**DOI:** 10.2196/65889

**Published:** 2025-04-09

**Authors:** Kathleen E Burch, Valerie L Tryon, Katherine M Pierce, Laura M Tully, Sabrina Ereshefsky, Mark Savill, Leigh Smith, Adam B Wilcox, Christopher Komei Hakusui, Viviana E Padilla, Amanda P McNamara, Merissa Kado-Walton, Andrew J Padovani, Chelyah Miller, Madison J Miles, Nitasha Sharma, Khanh Linh H Nguyen, Yi Zhang, Tara A Niendam

**Affiliations:** 1 Department of Psychiatry & Behavioral Sciences University of California-Davis Sacramento, CA United States; 2 Department of Psychiatry and Behavioral Sciences Weill Institute for Neuroscience University of California-San Francisco San Francisco, CA United States; 3 Department of Psychology University of California-Davis Davis, CA United States; 4 Institute for Informatics Washington University in St. Louis St. Louis, MO United States; 5 Herbert Wertheim School of Public Health and Human Longevity Science University of California San Diego San Diego, CA United States; 6 Center for Healthcare Policy and Research University of California-Davis Sacramento, CA United States

**Keywords:** eHealth, user-centered design, learning health system, psychosis, early psychosis, user-driven development, web-based, data visualization, surveys and questionnaires, measurement-based care

## Abstract

**Background:**

The Early Psychosis Intervention Network of California project, a learning health care network of California early psychosis intervention (EPI) programs, prioritized incorporation of community partner feedback while designing its eHealth app, Beehive. Though eHealth apps can support learning health care network data collection aims, low user acceptance or adoption can pose barriers to successful implementation. Adopting user-centered design (UCD) approaches, such as incorporation of user feedback, prototyping, iterative design, and continuous evaluation, can mitigate these potential barriers.

**Objective:**

We aimed to use UCD during development of a data collection and data visualization web-based and tablet app, Beehive, to promote engagement with Beehive as part of standard EPI care across a diverse user-base.

**Methods:**

Our UCD approach included incorporation of user feedback, prototyping, iterative design, and continuous evaluation. This started with user journey mapping to create storyboards, which were then presented in UCD workshops with service users, their support persons, and EPI providers. We incorporated feedback from these workshops into the alpha version of Beehive, which was also presented in a UCD workshop. Feedback was again incorporated into the beta version of Beehive. We provided Beehive training to 4 EPI programs who then piloted Beehive’s beta version. During piloting, service users, their support persons, and EPI program providers completed Beehive surveys at enrollment and every 6 months after treatment initiation. To examine preliminary user acceptance and adoption during the piloting phase, we assessed rates of participant enrollment and survey completion, with a particular focus on completion of a prioritized survey: the Modified Colorado Symptom Index.

**Results:**

UCD workshop feedback resulted in the creation of new workflows and interface changes in Beehive to improve the user experience. During piloting, 48 service users, 42 support persons, and 72 EPI program providers enrolled in Beehive. Data were available for 88% (n=42) of service users, including self-reported data for 79% (n=38), collateral-reported data for 42% (n=20), and clinician-entered data for 17% (n=8). The Modified Colorado Symptom Index was completed by 54% (n=26) of service users (total score: mean 24.16, SD 16.81). In addition, 35 service users had a support person who could complete the Modified Colorado Symptom Index, and 56% (n=19) of support persons completed it (mean 26.71, SD 14.43).

**Conclusions:**

Implementing UCD principles while developing the Beehive app resulted in early workflow changes and produced an app that was acceptable and feasible for collection of self-reported clinical outcomes data from service users. Additional support is needed to increase collateral-reported and clinician-entered data.

## Introduction

### Background

Research estimates that the lifetime prevalence of psychotic disorder diagnoses is approximately 1.5%, and the prevalence of psychotic symptoms is between 4.2% and 17.5% [1]. California, the most populous and second most diverse state in the United States [2], had a population of 39.11 million in 2023, according to the California Census Bureau. This indicates that 586,650 to 6.8 million Californians may experience psychosis symptoms in their lifetime. In response to this, many California counties have developed specialty early psychosis intervention (EPI) services, which vary widely in their implementation approach [3]. The Early Psychosis Intervention Network of California (EPI-CAL) [4] was developed to support the provision of quality EPI services and to create an infrastructure to conduct standardized measurement of the impact of early psychosis care delivery. To support this goal, the EPI-CAL team, in collaboration with several California counties, developed a learning health care network (LHCN) consisting of EPI programs across the state. The EPI-CAL LHCN later joined the national Early Psychosis Intervention Network [5], which allowed additional California EPI programs to participate. Members of the LHCN agreed to gather standardized information and outcomes from their clinics as part of measurement-based care (MBC). Collecting this information is critical to support quality early psychosis care provision within clinical programs as well as enhance statewide learning and development. For example, a narrative review of an MBC approach in behavioral health clinics found such benefits as significantly improving clinical outcomes, improving symptoms more quickly, and decreasing treatment costs [6].

To this end, the EPI-CAL team chose to design and implement a web-based and tablet app called Beehive. We chose the name Beehive to reflect the envisioned purpose for the app: to help LHCN programs learn and grow together for the betterment of the collective, just as bees work together to build a hive for the benefit of the colony. Beehive is a robust, stand-alone eHealth app for use by service users, their support persons, and EPI program providers. In this text, we use the term *service user* to refer to the individual with a psychosis diagnosis who is receiving mental health care from an early psychosis program. We use the term *support person* to refer to any person that the service user has chosen to involve in their care. This is typically the individual’s parent but might be another family member, a friend, a partner, or some other close relative. Beehive’s purpose is to promote MBC in EPI programs by standardizing data collection across a network of programs focusing on community partner priorities; supporting key components of care such as assessment, safety monitoring, and ongoing care delivery; supporting program-level management of care; and aggregating data across a large network to support evaluation and research at state and even national levels [4].

We chose an eHealth approach to implementing MBC due to its appropriateness for the EPI setting and its potential benefits. Despite the perceived challenges related to experiences of suspicion or paranoia, individuals experiencing mental health difficulties, such as schizophrenia and bipolar disorder, have widely found use of eHealth both feasible and acceptable [7-10]. Furthermore, the use of eHealth can support the advancement of MBC. For example, eHealth apps have been previously demonstrated to promote symptom and outcomes monitoring in both early psychosis care [11,12] and LHCNs [13]. Conducting MBC with eHealth enhances its benefits as it allows for data collection to be standardized across programs and instantly available. For example, MBC may promote collaboration across a care team [14-16], which is relevant for EPI programs for which the evidence-based treatment, coordinated specialty care, is inherently team based [17]. Use of eHealth to collect data in this setting allows data collected by one team member or entered by a service user to be instantly available for all team members. eHealth also enhances the benefits of MBC through data aggregation, which enables evaluation of program performance [16,18,19] or promotion of evidence-based treatments [16,20].

Though there are many potential benefits, there are also numerous barriers to implementation of both new eHealth technology and MBC. According to a systematic literature analysis, the top factors posing a barrier to eHealth app implementation include lack of digital health literacy, lack of devices, financing issues, service-user cognition, and security [21]. These barriers contribute to low adoption and user acceptance, which limit the success of implementation [22,23]. Barriers to implementation of MBC include training burden, concern that negative feedback causes harm to service users, and the time required for survey completion [24-26].

To pursue the benefits of using eHealth to implement MBC and mitigate the potential barriers, we developed Beehive with user-centered design (UCD) principles. UCD prioritizes the needs and expectations of the end user [27,28]. UCD approaches include dedicated design activities, active involvement of users in the design process, incorporation of their feedback, prototyping, and continuous evaluation [29], which can address low user acceptance and low adoption [30]. Beehive’s iterative development began with a collaborative process with service users, support persons, and EPI program providers to identify and prioritize which outcome measures should be collected in the app [31]. We also explored how service users, support persons, and EPI program providers wanted to be informed about data-sharing options in Beehive and built Beehive’s end user license agreement (EULA) workflow to incorporate user perspectives [32]. With the survey content and EULA workflow finalized, we moved onto the development of the user-facing parts of Beehive.

### Objectives

In this study, we aimed to (1) use UCD principles to create a co-designed web-based and tablet app, called Beehive, to support MBC in EPI programs, and (2) assess Beehive’s initial feasibility in clinical settings by piloting it in 4 EPI programs.

## Methods

### Design

**Figure 1:**
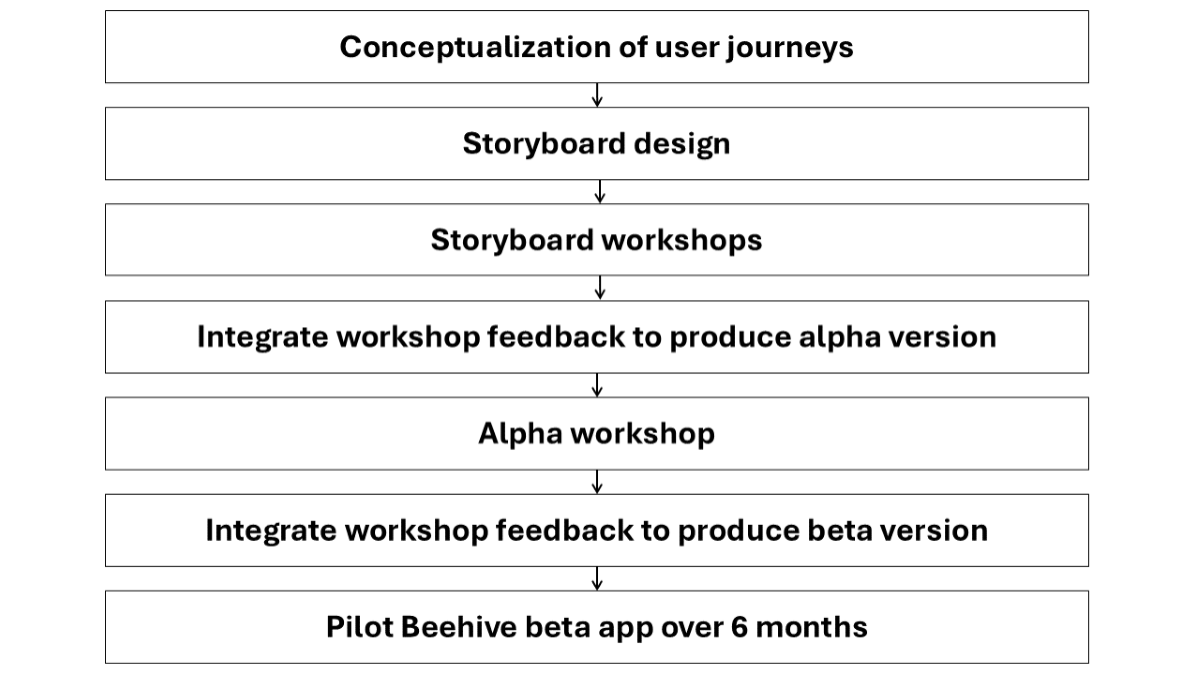
Study design for Beehive development.

To promote Beehive engagement across multiple types of users and across multiple domains of engagement, we integrated UCD principles of incorporation of user feedback, prototyping, iterative design, and continuous evaluation. Service users, support persons, and EPI program providers had multiple opportunities to provide feedback, which was incorporated throughout Beehive development. Figure 1 shows the study design from conceptualization through data collection.

The development process began with conceptualization of user journeys. User journey mapping envisions how specific types of users, such as a service user or a service provider, will interact with an app from access point through all required activities [33]. User journey mapping allowed us to identify which storyboards we should develop to present in UCD workshops and which user-types we needed to recruit for those workshops. Storyboards are a tool to visualize app workflows and the user interface [34]. We developed storyboards to present as prototypes in UCD workshops so that feedback could modify the app design before time was invested in coding the alpha version of the app. The alpha version of the app included core workflows and was both evaluated internally and presented in another UCD workshop to gather more feedback before coding the beta version of the app. The beta version of the app incorporated remaining feedback from storyboard workshops, new feedback from the alpha stage, and added the remaining core functionality that was not in-scope for the alpha version (eg, reports). The beta version of Beehive was piloted by 3 EPI programs over 6 months to further refine the app before launching it across all EPI-CAL programs. We used pilot data to assess initial use and uptake of Beehive’s beta version.

This UCD approach allowed the EPI-CAL team to receive and incorporate feedback during conception, design, and testing phases of eHealth app development, and include multiple perspectives to facilitate user engagement in eHealth. Notably, UCD has also been demonstrated to increase eHealth adoption and user acceptance in research and clinical settings [30,35].

### Participant Recruitment

#### UCD Workshops

For UCD workshops of the Beehive storyboard, we recruited participants from the following three EPI community partner groups: (1) EPI service users, (2) their support persons, and (3) EPI providers. Eligible participants were (1) actively or formerly affiliated with an EPI-CAL EPI program, (2) English speaking, and (3) able to provide written informed consent and assent (minors or conserved adults). EPI program providers were recruited through contact with the team lead of the 12 active EPI-CAL EPI programs. Service users and support person participants were invited either through EPI program provider referral or by the research team directly contacting individuals who had previously consented to be contacted for future research opportunities.

One EPI-CAL clinic agreed to participate in a workshop for the alpha version of the app to support the refinement of Beehive before piloting.

#### Piloting

In total, 4 EPI-CAL clinics agreed to participate in 6 months of Beehive beta testing before Beehive’s full launch across the entire EPI-CAL LHCN. During this period, programs integrated the Beehive app into standard clinical care. Pilot sites registered service users who were active in their program at the time of launching Beehive and new service users who started after the launch. Each participating program has different acceptance criteria for service users, and this has been described in a separate protocol paper for the EPI-CAL study (NCT04007510) [4]. Service users and support persons were excluded from piloting if they did not speak English because Beehive beta version was only available in English. At their first point of contact with Beehive, service users and primary support persons completed the Beehive EULA and were asked if they gave permission for their clinical data collected in Beehive to be used for research purposes [32]. We trained clinics to involve the legal guardians of service users aged <18 during EULA completion, and these service users were required to have a primary support person registered in Beehive. Individuals could update their data-sharing permissions at any time.

### Methods

#### User Journey Mapping

The EPI-CAL research team worked collaboratively with the contracted app developer in the user journey mapping and storyboard design phases for the Beehive tablet and web apps. Three primary user groups with distinct roles were identified: (1) service users or support persons, (2) direct-service providers, and (3) program administrators. Beehive user journeys were developed for each group.

For all user groups, user journeys were designed to guide individuals smoothly through Beehive onboarding and account creation to the EULA explainer video detailing the types of information Beehive collects, who can access their data, and how to select their preferred permissions for who can view their data [32]. Users then choose their data-sharing permissions.

Beehive then presents service users and support persons with a series of one-time and longitudinal surveys to measure clinical outcomes at 6-month intervals. Specific survey items associated with risk, such as suicidal or homicidal ideation, send real-time alerts to EPI program providers if they are endorsed by service users. Beehive creates visualizations of this survey data. While service users and support persons cannot independently access survey visualizations, we considered this is as part of their user journey because it should be shown to them by EPI program staff as part of regular care.

EPI program provider user journeys were designed to facilitate easy management of service user records and smooth navigation to service user data. A dashboard presents the most important information to users, such as outstanding survey alerts or other action items. A client list presents all registered service user records in list format with the most relevant information displayed. EPI program provider users can click into service user records to view survey results, survey visualizations, and complete provider-entered surveys. Providers can display survey results and survey visualizations as part of ongoing care with service users and their support persons to facilitate understanding and coordinate treatment priorities.

Administrator user journeys were designed to promote clinic-level management tasks, such as Beehive implementation and quality assurance. Administrator dashboards present aggregate-level information of survey data and allow for the ability to compare clinic averages to the average of the EPI-CAL LHCN. Administrator dashboards also present summaries of Beehive activity across the clinic. Administrator users can also download reports, including survey data reports, to use their clinic data for quality assurance or reporting requirements.

#### UCD Workshops

**Figure 2:**
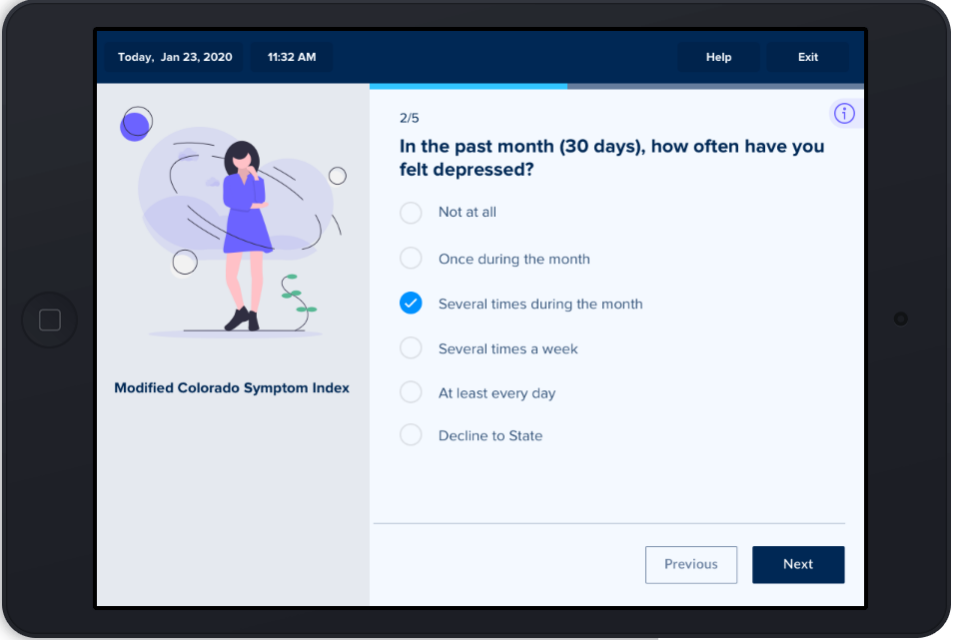
Survey item in storyboard.

**Figure 3:**
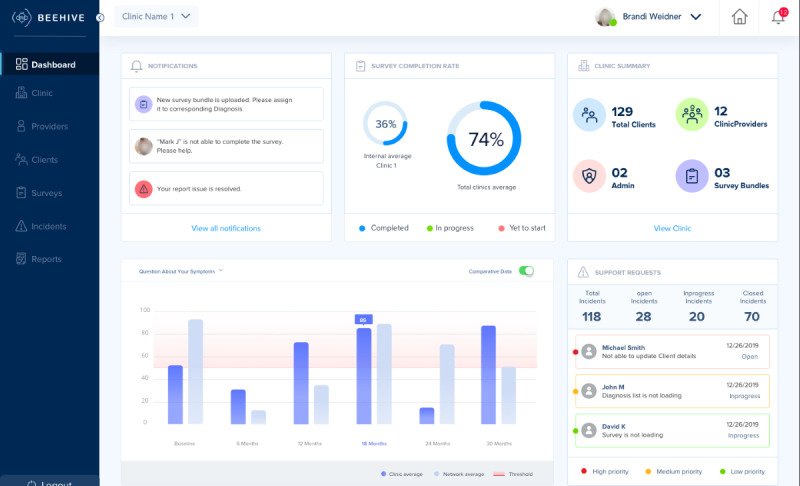
Clinical administrator dashboard in storyboard.

Next, the development team created dynamic storyboards of the above user journeys. These storyboards were presented to community partners from early psychosis clinics in 90-minute UCD workshops. Figures 2 and 3 are images from the storyboards presented to community partners.

In the storyboard workshops, we presented major features of the app and asked for feedback on the app’s look and feel, as well as functionality as it related to existing clinical workflow, and ease of use and acceptability for service users, their support persons, and EPI program providers. Because we determined 3 user-types during user journey mapping, we held 3 different types of storyboard workshops tailored to the journeys of these users: service users or support persons, direct-service providers, and program administrators. If EPI providers had roles in both direct-service provision and program administration, they could attend both groups. UCD workshops were conducted through videoconferencing to comply with the COVID-19 social distancing restrictions. Each session was audio recorded and included 2 facilitators (KEB, LMT, TAN, or SE) and a notetaker (VLT). There were no other individuals present other than researchers and participants. The notetaker took detailed notes, as close to verbatim as possible. Audio recordings were used as a reference to fix any portions of the notes which were not clear.

After storyboard workshops, we integrated feedback to make design changes to the alpha version of the app. During a 90-minute alpha testing workshop, we solicited feedback on the alpha app, with a special emphasis on how compatible it was with the existing clinical workflows. We created test accounts for each participant and had them complete various core workflows in the app, such as registering a service user, completing surveys, and reviewing data visualizations. This workshop included one facilitator (KEB) and one notetaker (LS). After the conclusion of all workshops, we continued to integrate feedback to make design changes in the beta version of the app.

#### Piloting

Finally, piloting of the beta version of Beehive was conducted over a 6-month period with EPI-CAL LHCN programs that were identified as pilot sites. We provided training to each pilot site and assigned them a point person from our research team to provide regular support, troubleshoot implementation challenges, and escalate app bugs and implementation barriers. We trained all program staff, regardless of clinical role. The training series showed users how to complete key Beehive workflows, introduced the EPI-CAL core assessment battery, and included activities on how to interpret data visualizations. We also met with key staff at each program to support them to devise a plan to integrate Beehive into their existing clinical workflows, such as how to integrate service user registration during the clinical intake process. The full description of this training and support is described in a separate paper [4]. During this training and support process, we received informal feedback from program staff about implementation successes and challenges.

**Figure 4:**
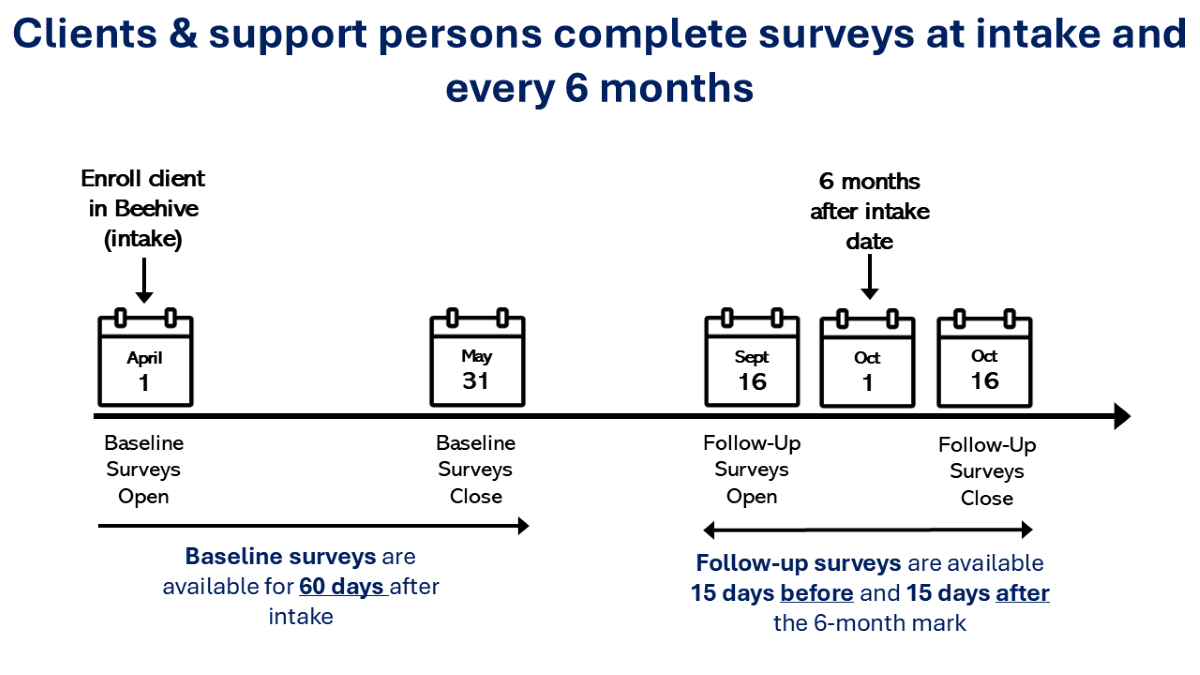
Beehive training slide showing survey windows during piloting.

During piloting, programs integrated the Beehive app into standard clinical care for all service users. Service users and their support persons were registered in Beehive web app by staff at their EPI program. Program staff entered the service users’ treatment start date which was considered the start of the service users’ baseline window in Beehive. At enrollment, baseline, and every 6 months thereafter, surveys were assigned to service users, their support person, and their provider. One-time surveys assessing lifetime experiences were available for all respondents at enrollment. Service users had 3 enrollment surveys, support persons had 1, and clinicians had 1. Longitudinal surveys were assessed at treatment baseline and every 6 months thereafter. Service users had 17 longitudinal surveys, support persons had 6 surveys, and clinicians had 9 surveys. The duration of baseline survey window was 60 days; the duration of follow-up survey windows was 30 days (15 days before and after the target completion date). Figure 4 shows a Beehive training slide with a visualization of survey windows.

Programs were instructed to enroll all service users, regardless of how long they had been affiliated with the EPI program. Therefore, some individuals may have been enrolled in Beehive after their baseline window had closed, and their first time point during piloting may have been a 6-month time point or a 12-month time point. Service users and support persons could access and complete surveys in-person at the clinic on the tablet app or they could complete them remotely via “web link.” The “web link” was a unique link that was texted or emailed to them weekly during survey windows if surveys were not fully completed. Surveys accessed via web link could be completed on any personal device that had access to the internet and a web browser. Service providers completed surveys and could review data on the web app. Baseline surveys were intended to support the clinical intake process, including initial assessment and collaborative treatment planning. Follow-up surveys were intended to support ongoing assessment, adjustments to treatment planning, and monitoring of treatment goals.

The EPI-CAL core assessment battery, including how it was created and all included measures, is described in a separate paper [4]. Briefly, Beehive survey content includes both the Early Psychosis Intervention Network core assessment battery [36] and additional measures based on EPI community partner feedback determined in earlier qualitative work for the EPI-CAL study [4,31]. A table of measures is included in the Multimedia Appendix 1 [4]. One survey of particular interest is the Modified Colorado Symptom Index (MCSI) [37], a measure central to the aims of the broader EPI-CAL study and which we asked sites to prioritize [4]. The MCSI is a 14-item, self-report scale which measures the frequency of psychiatric symptoms, including symptoms of mood, psychosis, cognition, forgetfulness, and risk to self and others. Respondents indicate frequency of symptoms over the past 30 days on a 0 to 4 scale of “not at all” to “at least every day.” Total scores range between 0 and 56, with higher scores indicating higher frequency and number of psychiatric symptoms.

### Data Analysis

In UCD workshops, we asked highly structured questions to solicit feedback on the storyboard and alpha version of Beehive. Subsequently, we organized our data categories relevant to the workflows and features we were evaluating. We then organized comments by whether they were supportive of the existing features or critical and asked for change so that we could focus on what features to move forward and what features to change as we created the alpha and beta versions of Beehive.

To investigate the initial feasibility of Beehive in EPI-CAL clinics, we reviewed descriptive statistics of pilot participants, including registration, enrollment, participant characteristics, and survey completion. Engagement with surveys and survey completion were examined in three ways as follows: (1) determine the proportion of service users for whom any data were entered, regardless of respondent type, (2) determine the rate of survey completion across all available surveys, and (3) evaluate whether participants completed all, partial, or no surveys across survey time points during piloting phase. Partial survey completion indicates that the respondent completed at least 1 survey, but did not complete all of their surveys in the specified time point. We also evaluated completion of MCSI because it is a measure that we asked programs to prioritize.

### Ethical Considerations

The institutional review board of the University of California, Davis, approved the study (1403828-21, California Collaborative Network to Promote Data-Driven Care and Improve Outcomes in Early Psychosis). In addition, several of the counties and universities with a program participating in EPI-CAL required a separate review and approval of the project by their institutional review board. All study participants provided written informed consent and assent (as appropriate). Participants received US $30 compensation for each workshop they participated in. Participants in the piloting phase were not compensated because integration of Beehive was part of routine care in the EPI program. Audio recordings UCD workshops include voice print identifiers and are stored in compliance with University of California, Davis HIPAA (Health Insurance Portability and Accountability Act) policies and procedures. Data collected during piloting for research includes limited identifiers including zip code, dates of service, and month and year of birth. Only trained research staff with a need-to-access have access to identifiable data.

## Results

### UCD Workshops

We conducted 14 storyboard workshops with 77 total participants between April 3, 2020, and August 28, 2020. In total, 4 workshops were with service users (n=8, 10%) and their support persons (n=9, 12%). In addition, 10 workshops were with EPI program providers (n=60, 78%), including 6 for service providers, 3 for administrators, and 1 for both service providers and administrators. Demographics for workshop participants are provided in Table 1.

We completed an interim analysis of storyboard workshop data in May 2020. We completed the final analysis of workshop data in August 2020, after all groups were completed. After each analysis, we discussed and synthesized the feedback for the developers to support app development. We attempted to balance the needs of all types of participants. However, if there were needs or feedback in direct contrast with one another, we prioritized service-user feedback due to our value of centering service-user feedback in this app. This feedback and the action taken to address it are summarized in Table 2.

**Figure 5:**
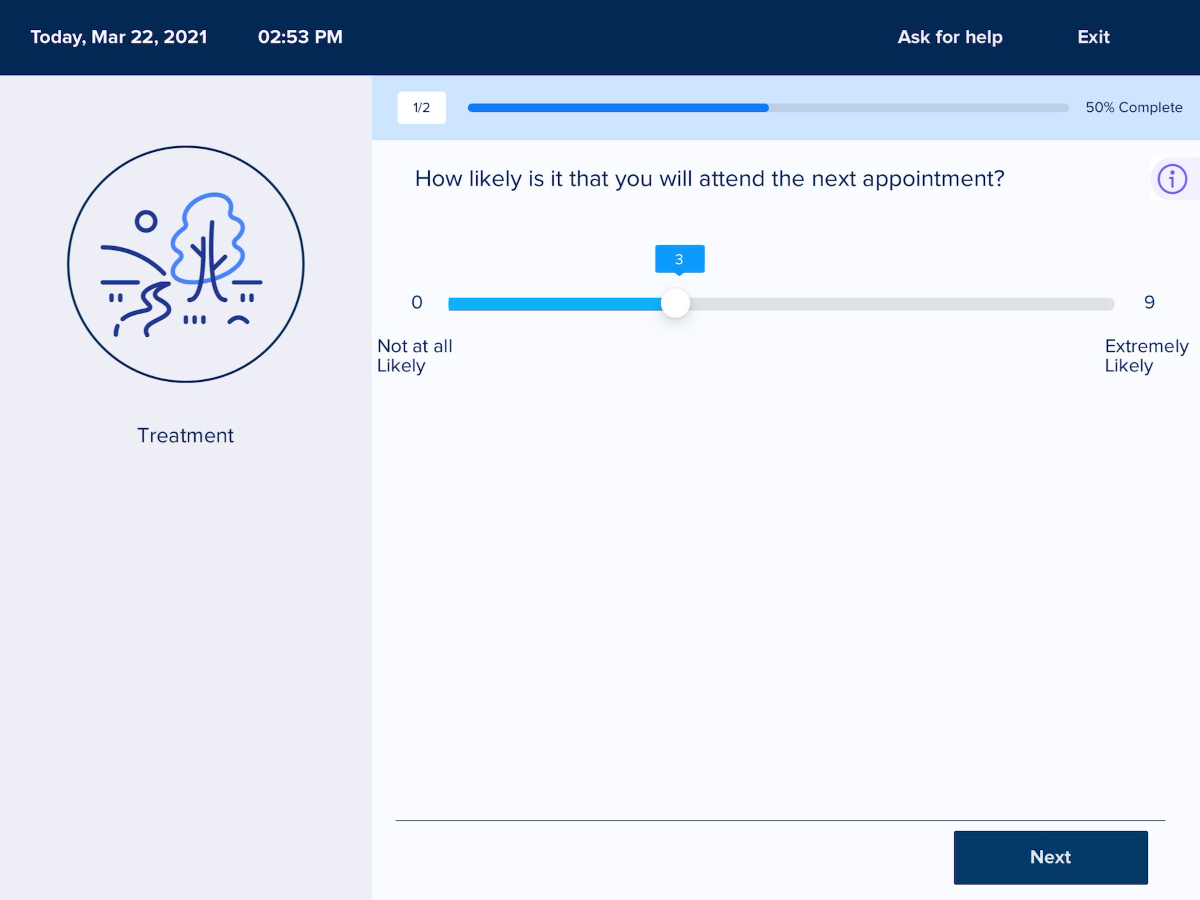
Survey item in beta.

**Figure 6:**
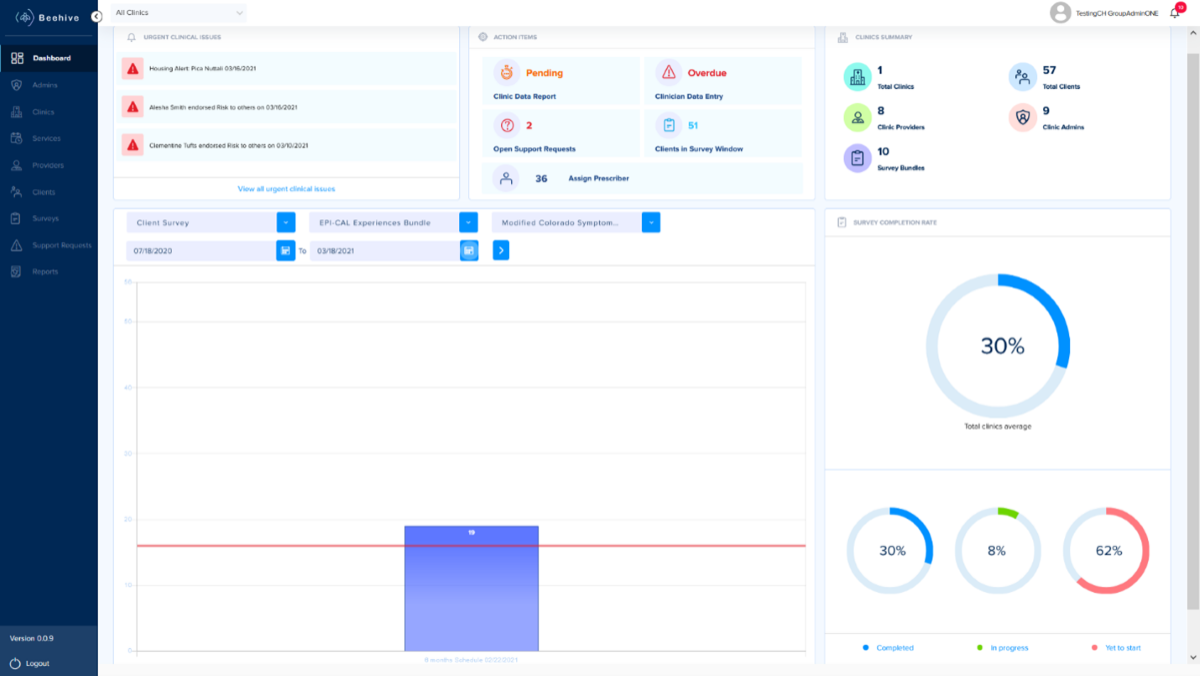
Clinical administrator dashboard in beta.

We conducted 1 alpha workshop in October 2020 with 4 EPI program provider participants. Feedback from this workshop was analyzed in October 2020. During this workshop, participants identified a few bugs in the app, but their feedback primarily focused on ideas for integrating Beehive into clinical workflows. For example, they suggested that Beehive training should include best practices for how providers can review the data, engage with the data, and make the most out of Beehive. They also shared concerns about using technology in telehealth settings. For example, switching to telehealth in response to the COVID-19 pandemic had been very difficult for some families, and they predicted those same families would find using Beehive challenging if the clinic could not meet with them in-person to teach them how to use it. They were less concerned about service users and support people using Beehive on a tablet in the clinic where they could provide in-person support. Finally, participants brought up the importance of shifting the culture of clinics to view data collection as an important part of treatment, not just an extra task where information is being extracted from service users. For example, surveys should be directly related to service-user recovery goals. Participants discussed how visualizations could be used to demonstrate the clinical utility of gathering these data. For example, 1 clinician said they would want to use the graphs to point out the way a service user is improving or doing better and that they would want to highlight their strengths. Another participant cautioned that some service users may not want to look at data visualizations and that this should be an optional part of their care. Figures 5 and 6 illustrate design changes present in the beta version of app after the conclusion of all workshops.

**Table 1:** Demographics of user-centered design workshops^a^.

		Service users (n=8)	Support persons (n=9)	EPI^b^ program providers (n=60)
**Clinic type, n (%)**				
	Medi-Cal	7 (88)	6 (67)	30 (50)
	Private insurance	<5 (<63)	<5 (<55)	30 (50)
Age (y), mean (range)		22.50 (16-33)	41.50 (14-60)	36.25 (26-50)
**Sex at birth, n (%)**				
	Female	<5 (<63)	8 (89)	43 (72)
	Male	6 (75)	<5 (<55)	17 (28)
**Gender, n (%)**				
	Female	<5 (<63)	8 (89)	43 (72)
	Male	6 (75)	<5 (<55)	16 (27)
	Nonbinary	<5 (<63)	—^c^	—
	Missing	—	—	<5 (<8)
**Race, n (%)**				
	African or African American or Black	<5 (<63)	—	5 (8)
	Asian	—	—	6 (10)
	White or Caucasian	<5 (<63)	6 (67)	33 (55)
	Other	<5 (<63)	<5 (<55)	10 (17)
	More than one	<5 (<63)	<5 (<55)	<5 (<8)
	Missing	—	<5 (<55)	<5 (<8)
**Ethnicity, n (%)**				
	Latinx	<5 (<63)	5 (56)	26 (43)
	Not Latinx	5 (63)	<5 (<55)	33 (55)
	Missing	—	—	<5 (<8)
**Sexual orientation, n (%)**				
	Bisexual or gay or lesbian	<5 (<63)	—	5 (8)
	Heterosexual	5 (63)	9 (100)	54 (90)
	Other	<5 (<63)	—	<5 (<8)

^a^Cells with fewer than 5 individuals are masked to protect the identity of participants.

^b^EPI: early psychosis intervention.

^c^Not available.

**Table 2:** Implementation of feedback from user-centered design workshops.

Problem or need identified in workshop	Solution implemented in alpha version
The color scheme and layout seemed “overly clinical”	Brought in more color into the palette and added icons for visual information
Some important aspects of the user-interface were too subtle, such as the survey progress bar or the urgent clinical issues widget	Changed color and design to make it more prominent
Service-user and support person registration were only available as self-registration and could not be completed by EPI program providers	Added this workflow to the web app so that EPI program providers may complete it in advance of service users engaging with surveys
Clinic-level data for service-user demographics was not visualized	Added clinic-level visualizations for race, ethnicity, sex, gender identity, and other demographic metrics
Not all service users or support persons wanted to see score thresholds or comparative data on clinical measures, but some did	Added a toggle to individual-level visualizations so that users can turn on the threshold information or comparative data if they want to see it, or turn it off if they do not want to see it
Service users might have differed on which individual-level survey visualization they wanted to see by default on their data view page	Added feature that allows users to set which measure displays by default for each service user
Some language used in the app needed to be clarified for users to understand what data was being collected or how certain features worked	Changed “homeless” to “without a permanent address” when assessing housing status; changed “help” to “Ask for help” to make it clearer that selecting button will alert the EPI program provider; changed “Diagnosis” to “Primary diagnosis”
Different programs used different words to refer to service users, and individuals might have varied on their preference for what word to use regardless of what their program tended to use	Wherever possible, implemented dynamic text so the service user’s preferred name shows throughout the app, rather than any specific word to denote “service user”
Program staff wanted to see overall progress on completion of all surveys at any given time point	Added in visual indicator to show survey completion across multiple surveys (not just while completing one individual survey)
When visualizing a survey, they wanted to have more than just the global score visualized. Also, they wanted a visualization that showed responses to individual items	Added in a visualization that shows individual items as well as the global score
Service users and support persons might not have preferred the official names for measures and might have preferred a more simplified title	Added the ability to enter a display name for surveys (eg, “Family Impact” instead of “Burden Assessment Scale”)
Early psychosis intervention program providers needed a way to see both the official measure name as well as the display name	Added a hover modal on survey titles to show the display name for the survey
The provision of clinic services might have been fully remote for the foreseeable future, and the current design of Beehive only allowed service users and support persons to complete surveys on a tablet in-person at the clinic	Design a web link solution which allows service users and support persons to answer surveys remotely. A link to complete their surveys can be emailed or texted to them

### Piloting

We conducted piloting of Beehive beta app between March 2021 and September 2021. Our training and ongoing support of pilot sites allowed us to gather informal feedback about both the training and the Beehive app. We used this feedback to make adjustments in real time, when possible, or to plan for future changes to Beehive.

We made real-time changes to the training approach in response to program needs based on our observations and their feedback. At the time of training pilot sites in early 2021, these EPI programs were navigating constant uncertainty related to the COVID-19 pandemic, including influx of service users, uncertainty about work location, reduced workforce, etc. In response to this environment, we found it necessary to ask sites to focus on small implementation steps even though we trained them on all available workflows. For example, we asked pilot sites to initially focus on engaging service users and their support persons to complete enrollment and complete surveys. When that was mastered, we asked them to focus on engaging new service users and support persons with Beehive during their clinical intake process. Finally, toward the end of the piloting period, we encouraged them to focus on entering EPI program provider-entered data. Even if pilot sites registered existing service users, they were asked to set the service user’s survey baseline date to align with their start in the EPI program. Therefore, some service users may have never been assigned surveys during their baseline survey window.

During the piloting phase, 93 service users, 78 support persons, and 86 service providers were registered across 4 clinics. Of the 93 service users who were registered into Beehive by their program, 59 (63%) completed the Beehive EULA during piloting, including 48 (51%) individuals who gave permission to use their data for research. Of the 78 support persons registered to a service user in Beehive, 52 (66%) completed the Beehive EULA, including 42 (54%) individuals who gave permission to use their data for research. Of these 42 support persons, 5 were excluded from analysis because the service user they were registered with did not give permission to use data in research, and we prioritize the data-sharing decision of the service user regarding use of collateral data for research purposes.

**Figure 7:**
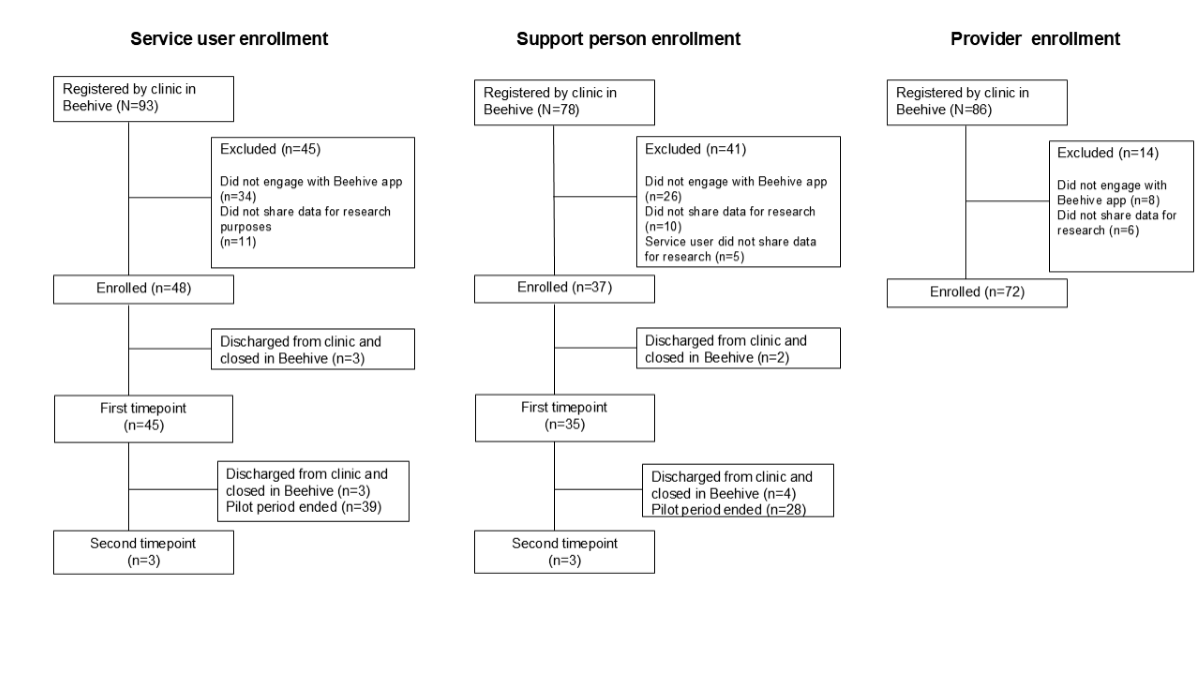
Registration and enrollment during Beehive piloting.

While most users entered at least one survey window during the piloting phase, 3 were discharged from their program before longitudinal surveys were available. Of the 86 service providers registered, 78 (91%) completed the Beehive EULA, including 72 (84%) individuals who gave permission to use their data for research. This information is presented in Figure 7.

Participant demographics and EPI program provider professional background are provided for individuals who agreed to share their data for research purposes in Tables 3 and 4. Of note, age is missing for some EPI program providers and support persons. This data was collected during registration, and this field was not included in the first release of the beta version of the app. Therefore, some users were not able to complete this field during registration and did not return later to update it, yielding missing data for 15 (36%) support persons and 37 (51%) EPI program providers.

First, to examine engagement during piloting, we assessed the number of service users that had entered any data that could be used in care. During piloting, respondents entered survey data for 85% (n=41) of service users. This includes self-reported data for 75% (n=36), collateral-reported data for 42% (n=20), and EPI program provider–entered data for 17% (n=8).

Second, we evaluated survey completion across the total amount of available surveys. A total of 1517 surveys were assigned across all respondent types during piloting and 35.4% (n=537) of those surveys were completed across all time points. We also evaluated survey completion by respondent type. Across all time points, service users completed 396 (49.4%) of 802 assigned surveys. Support persons completed 113 (47.5%) of 238 assigned surveys. EPI program providers completed 28 (5.9%) of 477 assigned surveys.

Finally, we evaluated how many respondents completed all, partial, or no surveys at each time point. Because participants could be enrolled at any point in treatment, the first time point for a service user may not have been their “baseline” appointment. These data are presented in Table 5.

The MCSI was completed by 54% (26) of service users, and 7 were excluded for responses of “prefer not to say” (Total Score: mean 19.58, SD 16.81). Additionally, 35 service users had a support person who could complete the Modified Colorado Symptom Index, and 56% (n=19) of support persons completed it. From these 19, 5 were excluded for responses of “prefer not to say” (mean 26.71, SD 14.43).

Through piloting, we also gathered informal feedback about workflows that could be improved in Beehive and that we would address with future change orders to the app after the testing of the beta version of Beehive. For example, we received feedback that the survey windows, initially chosen to mirror the data collection windows of clinical trials, were far too narrow and restrictive for data collection in community mental health programs. Program staff users also indicated that they wanted a better way of seeing a summary of what surveys service users and support persons completed. Service user and support person feedback was relayed to our team via program staff. For example, service users and support persons wanted to customize the day and time they received the web link via SMS text messaging and email.

**Table 3:** Demographics of Beehive pilot participants^a^.

			Service users (n=48)		Support persons (n=42)		EPI^b^ program providers (n=72)
Age (y), mean (range)			18.88 (12-31)		44.11 (31-61)^c^		33.89 (22-50)^d^
**Sex at birth, n (%)**							
	Female	24 (50)		21 (70)		30 (42)	
	Male	24 (50)		6 (20)		5 (7)	
	Prefer not to say	—^e^		<5 (<12)		—	
	Missing	—		—		37 (51)	
**Gender, n (%)**							
	Female	18 (38)		21 (70)		30 (42)	
	Male	22 (46)		6 (20)		5 (7)	
	Nonbinary	<5 (<10)		—		—	
	Questioning or unsure of gender identity	<5 (<10)		—		—	
	Prefer not to say	<5 (<10)		<5 (<12)		37 (51)	
**Race, n (%)**							
	African or African American or Black	15 (31)		7 (17)		5 (7)	
	American Indian or Alaskan native	<5 (<10)		—		—	
	Asian	<5 (<10)		<5 (<12)		8 (11)	
	Hispanic or Latinx only	10 (21)		<5 (<12)		19 (26)	
	White or Caucasian	10 (21)		10 (24)		33 (46)	
	More than one race	8 (17)		<5 (<12)		5 (7)	
	Other	—		—		<5 (<7)	
	Prefer not to say	<5 (<10)		<5 (<12)		—	
	Unsure	—		<5 (<12)		—	
	Missing	—		12 (29)		<5 (<7)	
**Ethnicity, n (%)**							
	No—I do not identify as Hispanic or Latinx	27 (56)		17 (40)		41 (57)	
	Yes—I identify as Hispanic or Latinx	14 (29)		5 (12)		28 (39)	
	Unsure or do not know	5 (10)		5 (12)		—	
	Prefer not to say	<5 (<10)		<5 (<12)		<5 (<7)	
	Missing	—		12 (29)		<5 (<7)	
**Service user diagnosis, n (%)**							
	Clinical high risk	—		—		—	
	Attenuated psychosis symptoms	6 (13)		—		—	
	First episode psychosis	—		—		—	
	Substance induced psychotic disorder with onset during intoxication	<5 (<10)		—		—	
	Mood disorders with psychotic features	9 (19)		—		—	
	Schizoaffective disorder (bipolar or depressive type combined)	10 (21)		—		—	
	Schizophrenia	5 (10)		—		—	
	Other specified schizophrenia spectrum disorder	<5 (<10)		—		—	
	Unspecified psychosis	<5 (<10)		—		—	
	Other first episode psychosis	7 (15)		—		—	
	Clinical high risk or first episode psychosis status not confirmed	—		—		—	
	Anxiety disorders	<5 (<10)		—		—	
**Number of support persons registered in Beehive, n (%)**							
	None	14 (29)		—		—	
	1	29 (60)		—		—	
	2	5 (10)		—		—	
**Relationship of support persons with service user, n (%)**							
	Parent (adoptive)	—		5 (12)		—	
	Parent (biological)	—		34 (81)		—	
	Stepparent	—		<5 (<12)		—	
	Spouse or partner	—		<5 (<12)		—	
	Sibling	—		<5 (<12)		—	

^a^Cells with less than 5 individuals are masked to protect the identity of participants.

^b^EPI: early psychosis intervention.

^c^Data missing for 15 individuals.

^d^Data missing for 37 individuals.

^e^Not available.

**Table 4:** Professional background of early psychosis intervention program providers registered during Beehive pilot (N=72)^a^.

Background			Values, n (%)
**Education level**			
	HS^b^ diploma or GED^c^	7 (10)	
	Associate’s degree	<5 (<7)	
	BA^d^ or BS^e^	16 (22)	
	MA^f^ or MS^g^	18 (25)	
	MFT^h^	7 (10)	
	MSW^i^	<5 (<7)	
	PsyD^j^	5 (7)	
	PhD^k^	6 (8)	
	MD^l^	9 (13)	
**Professional role**			
	Administrative support staff	<5 (<7)	
	Case manager or recovery coach	<5 (<7)	
	Clinic coordinator	6 (8)	
	Clinical supervisor or team lead	<5 (<7)	
	Clinician or therapist	30 (42)	
	Family advocate	<5 (<7)	
	Peer support specialist	<5 (<7)	
	Prescriber or psychiatrist or Other medical personnel	9 (13)	
	Program director	<5 (<7)	
	Research staff	<5 (<7)	
	Supported education and employment specialist	<5 (<7)	
	Other	7 (10)	
**Licensure status**			
	Unlicensed	49 (68)	
	Licensed	23 (32)	
**Years licensed (n=23)**			
	≤1	8 (38)	
	1 to 6	7 (33)	
	≥7	6 (29)	
**Number of languages in which services are provided**			
	1	47 (65)	
	2	18 (25)	
	Missing	7 (10)	
**Languages for service provision^m^**			
	English	60 (92)	
	Spanish	18 (25)	
	Arabic	<5 (<7)	
	Hmong	<5 (<7)	
	Tagalog	<5 (<7)	
	Other	<5 (<7)	

^a^Cells with less than 5 individuals are masked to protect identity of participants.

^b^HS: high school.

^c^GED: general educational development.

^d^BA: Bachelor of Arts.

^e^BS: Bachelor of Science.

^f^MA: Master of Arts.

^g^MS: Master of Science.

^h^MFT: Master of Marriage and Family Therapy.

^i^MSW: Master of Social Work.

^j^PsyD: Doctor of Psychology.

^k^PhD: Doctor of Philosophy.

^l^MD: Doctor of Medicine.

^m^Respondents could select more than one response, so percentages will be greater than 100%.

**Table 5:** Survey completion by respondent type^a^.

		Service users, n (%)	Support persons, n (%)	EPI^b^ program providers, n (%)
**Survey completion at enrollment^c^**				
	All	30 (63)	17 (50)	10 (21)
	Partial	5 (10)	—^d^	—
	None	13 (27)	17 (50)	38 (79)
**Survey completion at first time point^e^**				
	All	18 (40)	17 (55)	—
	Partial	8 (18)	3 (10)	4 (9)
	None	19 (42)	13 (42)	41 (91)
**Survey completion at second time point^f^**				
	All	1 (33)	—	—
	Partial	—	—	—
	None	2 (67)	2 (100)	3 (100)

^a^Partial survey completion indicates that respondents completed at least one survey, but did not complete all assigned surveys.

^b^EPI: early psychosis intervention.

^c^Total respondents for service users, n=48; support persons, n=34; and service providers, n=48.

^d^Not available.

^e^Total respondents for service users, n=45; support persons, n=31; and service providers, n=45.

^f^Total respondents for service users, n=3; support persons, n=2; and service providers, n=3.

## Discussion

### Principal Findings

This study describes the EPI-CAL program’s design and acceptability testing approach for a custom web-based and tablet app, Beehive, to support systematic data collection, care delivery, program evaluation, and research across a statewide network of EPI programs. Our goal was to develop an app that was clinically useful for, usable by, and acceptable to diverse EPI programs across the state of California.

To ensure the app best matched the needs of the EPI participants, we adopted a UCD approach to develop Beehive. Previous research in the mental health digital space supports that active involvement from the app’s intended users during the development phase can improve the appropriateness of the end product for the users of interest [38]. Initial feedback across the 3 development phases was primarily collected in workshops (storyboard and alpha version) and during pilot implementation (beta version). In storyboard and alpha workshops, we presented prototypes to demonstrate major features of the app and asked for feedback on the app’s “look and feel,” compatibility with existing clinical workflow, and ease of use and acceptability for service users, their support persons, and EPI program providers. Consistent with other studies who have included end users during the design phase of their eHealth apps [38-41], feedback from these workshops resulted in immediate changes to the alpha and beta apps that would not have otherwise been made.

During piloting, we continued to collect user feedback around Beehive features, as well as assess acceptability of the app by examining preliminary enrollment and survey completion. Our enrollment and survey completion rates are consistent with the acceptability of other mental health apps developed using a UCD approach [42,43], although there is wide variability depending on the implementation approach.

During the design and testing phase, we observed that different types of community partners expressed different, and at times conflicting, needs. For example, we asked participants about their preferences for seeing score thresholds or comparative data as part of the visualization for their clinical measures. Some service users said that, in times of relapse or increasing intensity of symptoms, additional information on the visualization would be demoralizing. In contrast, many participants could imagine scenarios where that information would be useful as a form of psychoeducation to normalize service-user experiences or understand the relative severity of symptoms. To address these diverse needs and promote engagement with Beehive we added a toggle to individual-level visualizations so that users can turn the threshold information or comparative data on or off. A flexible design approach that is tailored to an individual’s needs has been shown to be more efficacious in a mobile health app setting [43,44]. Therefore, design changes incorporated flexibility where possible to enable our team to meet the various needs of individuals while maintaining consistent implementation to meet evaluation and research goals.

Similarly, user feedback informed our training approach. For example, some EPI program provider users expressed that they would use the graphs in the app in clinical care with service users to highlight strengths and progress. In contrast, another EPI program provider participant cautioned that some service users may not want to look at data visualizations and that this should be an optional part of their care. Thus, our training approach highlights how visualizations may be used in direct care but are not prescriptive. Feedback from workshop participants also highlighted the importance of shifting clinic culture to view data collection as a key part of care provision. Our team considers EPI program providers to be integral in promoting engagement for service users and their support persons as it is the EPI program providers who communicate why Beehive is being used in care. To begin addressing potential barriers of buy-in and engagement for all users, we designed our trainings to include the context of why Beehive and MBC were being implemented in their programs, including a presentation on the potential value of Beehive (designed and delivered by author LS) [45].

During piloting, we observed barriers to integration. For example, despite our designing the first training such that the programs could start registering service users immediately afterward, the programs failed to do so. When asked, programs informed us that they were nervous to receive questions about Beehive that they did not know how to answer. This resulted in our team creating materials to provide more structure for programs as they introduced Beehive to service users, such as an introduction script, Beehive infographics, and other handouts. Once programs started enrolling existing service users, many found it difficult to transition enrolling new service users, given that they already had numerous documentation requirements during their clinical intake process. In response, we added a “workflow meeting” to our training series where we asked program leadership and key staff involved in intakes to walk us through their existing procedures so that we could help brainstorm where the required Beehive workflow steps could be implemented and who from their program would be responsible for each step. We additionally observed that clinician-entered data were hard for sites to prioritize. For example, there was a lack of clarity within teams about who was responsible for entering these data and what training was required. Therefore, we chose to add another “workflow meeting” between key program staff and our team to help programs identify who was responsible for which surveys, who needed training, and how programs could monitor survey completion. We added these workflow meetings to our formal training protocol and made the support materials available to all sites who joined after the piloting phase.

Furthermore, our team worked with programs beyond the piloting phase to ensure that we continued to incorporate individual feedback and offer continuous support, which is key to successful adoption and can improve engagement [46]. After the piloting of the beta version of Beehive concluded, we continued to make development changes to meet users’ needs, such as further design changes to the admin dashboard, widening survey completion windows, adjusting and eventually allowing customization of the frequency and timing of web link notifications, allowing the EULA to be completed before survey baseline date, simplifying registration fields, adding a survey status page, adding additional survey visualizations, adding a workflow for providers to enter data collected outside of Beehive, and prioritizing the order of additional languages in the app based on active need in the participating clinics. This iterative approach in response to user feedback is consistent with the development process of other eHealth apps [47].

This study highlights how critical it is for programs using a continuous improvement approach, such as UCD, to budget appropriately for ongoing development needs and staff time for ongoing support. As long as an app is in use and collecting data from real users, there should be a plan for ongoing project management and app development to address feedback from users, improve engagement and useability, and respond to changing needs. Implementing UCD from the outset allowed our team to be aware of and address user concerns before investing valuable time and resources in initial development and implementation. Focusing on workflow during the storyboard and alpha phases of app and continuing this collaborative relationship throughout the implementation phase resulted in an app that represents the interests and needs of users.

During the piloting, we observed that survey completion rates varied among different types of users. This variance may be partially explained by our training approach during piloting (see the Results section). As we continue to collect data after the piloting phase, we can evaluate if this trend continues beyond the initial onboarding period throughout multiple years of data collection. These varied results may also reflect the challenges of implementing MBC, with or without an eHealth app, such as the training burden and limited time to conduct new duties associated with eHealth implementation [24-26,31]. When implementing outcomes data collection in these settings, it may be critical to gather only the minimum required data from EPI program providers (eg, diagnosis) and rely on service-user self-report measures whenever possible. Future analyses will examine the relationship between characteristics of EPI program providers (eg, degree and years licensed) and completion rates of clinician-entered data. Future work, including barriers and facilitators interviews with users after they gain more experience using Beehive, will be used to prioritize the needs and perspectives of our users in the ongoing development of Beehive and to better understand the reasons why users do or do not engage with the app [48].

### Limitations

The COVID-19 pandemic introduced multiple challenges for our study, which may have reduced the breadth and diversity of participation in various phases of the project. We offered workshops over remote teleconferencing instead of in-person, which may have excluded individuals who are less comfortable with using technology. This may have disproportionately impacted on the recruitment of service users and support persons for participation in workgroups, as our participant numbers were lower than previous studies where we were conducting in-person research [31]. To reduce bias that may have resulted from this imbalance, we chose to prioritize the feedback of service users and support persons if there was conflicting feedback between these participants and EPI program provider participants. In addition, the beta and full versions of Beehive have been introduced to all service users in participating programs, regardless of their comfort with technology, and this has allowed us to incorporate informal feedback from these individuals as we have continued to make improvements to Beehive.

Much of our data in UCD workshops were gathered at the start of the COVID-19 pandemic, before anyone had experienced the long-term shift in daily practices brought on by the increased use of telehealth and remote working. We sought feedback on an app that was intended for in-person use and received feedback based on participants’ experience of using in-person services. This highlights the importance of planning multiple opportunities for soliciting and incorporating feedback from sites so that apps can be responsive to changing environments.

Our workshops and piloting were only conducted in English. To serve the diverse population of California, Beehive needs to be both translated and adapted, a process known as localization [49], into at least 15 languages. Since Beehive’s launch, we have localized into 7 additional languages. We continue to solicit feedback from users, including those whose primary language is not English, to inform the ongoing development of Beehive, and we will continue localize this app into additional languages.

While we used our prior knowledge of mental health development in the development of Beehive [11,12], we did not use a structured analysis approach for the feedback obtained during workshops due to time constraints imposed by project deliverables. To reduce the impact of subjective biases, the researchers who conducted each group debriefed afterward to review the notes, and recordings were referenced if notes were unclear or vague. In addition, all decisions about how to incorporate feedback from these notes into app development were made collaboratively by authors KEB, LMT, TAN, and VLT. Future work in this area will benefit from organized approaches to data collection and formal qualitative analysis [50,51].

### Conclusions

Working with community partners to co-design an eHealth app for use in community EPI programs helped us to anticipate and resolve barriers earlier in the app development and implementation pipeline. On the basis of our observation and the data, there appeared to be high levels of engagement with Beehive. This resulted in feedback and continued design improvements which allowed our team to be better poised to launch Beehive across the EPI-CAL LHCN. Variance in survey completion rates among respondent types suggests that support persons and EPI program providers especially may need additional support.

## Appendix

Multimedia Appendix 1 The Early Psychosis Intervention Network of California outcomes collected in Beehive.

## Abbreviations

EPI: early psychosis intervention
EPI-CAL: Early Psychosis Intervention Network of California
EULA: end user license agreement
HIPAA: Health Insurance Portability and Accountability Act
LHCN: learning health care network
MBC: measurement-based care
MCSI: Modified Colorado Symptom Index
UCD: user-centered design

## Data Availability

The datasets generated and analyzed during this study are available from the corresponding author on reasonable request.
