# Supplementary material for: Co-Designing a Web-Based and Tablet App to Evaluate Clinical Outcomes of Early Psychosis Service Users in a Learning Health Care Network: User-Centered Design Workshop and Pilot Study
Source: JMIR Hum Factors. 2025 Apr 9;12:e65889. doi: 10.2196/65889 (PMC12018865; doi:10.2196/65889)
Supplement: Multimedia Appendix 1 [file humanfactors_v12i1e65889_app1.docx]

### Appendix 1. EPI-CAL Outcomes Collected in Beehive

This table is included with permission from the original authors[[1]](https://sciwheel.com/work/citation?ids=17212680&pre=&suf=&sa=0&dbf=0).

| **Domain** | **Respondent** | **Measure and/or Source^a^** | **Timepoint** |
| --- | --- | --- | --- |
| Demographics & Background | Service user | - EPI-CAL team | Enrollment |
| Demographics and Background | Service user | - EPI-CAL researchers  - California State Required Demographics Reporting  - Modification from EPINET version of this question "Are you a veteran?" required question for PEI/INN Reporting  - A question measures a risk factor for persistent poverty(29).  - An item was created by the EPI-CAL team and assesses factors which put a person at increased risk for homelessness (30)  - Part of EPINET CAB(26) (https://nationalepinet.org/core-assessment-battery-cab/) | Enrollment |
| Primary Caregiver background | Service user | - EPI-CAL researchers and EPINET CAB(26) | Enrollment |
| Traumatic Events and Experiences | Service user | - Pediatric Adverse Childhood Experiences (ACEs) and related life events screener (PEARLS) (31) | Enrollment |
| Demographics and Background | Service user | - A question measures a risk factor for persistent poverty(29).  - An item was created by the EPI-CAL team and assesses factors which put a person at increased risk for homelessness, as described in literature (30)  - Part of EPINET CAB(26) | Every 6 months (including Baseline) |
| Education | Service user | - Homelessness Risk item created by EPI-CAL team derived from literature review (30) | Every 6 months (including Baseline) |
| Employment and Related Activities | Service user | - EPI-CAL researchers and EPINET CAB(26) | Every 6 months (including Baseline) |
| Social Relationships | Service user | - Attachment Item from Social Provisions Scale (32)  - EPI-CAL researchers  - Distress Disclosure Index (33) | Every 6 months (including Baseline) |
| Family Functioning | Service user and PSP | - SCORE-15 Index of Family Functioning and Change (34) | Every 6 months (including Baseline) |
| Legal Involvement and Related | Service user | - EPI-CAL researchers and EPINET CAB(26). Response options were informed from literature (35) and community partner feedback during focus groups(25). | Every 6 months (including Baseline) |
| Substance Use | Service user | - EPINET CAB | Every 6 months (including Baseline) |
| Medication, Side Effects, and Treatment Adherence | Service user | - Adherence Estimator  - Glasgow Antipsychotic Side-effect Scale (GASS) (36)  - Brief Adherence Rating Scale (BARS) (37)  - Additional items derived from focus group feedback and written by EPI-CAL team | Every 6 months (including Baseline) |
| Intent to Attend and Complete Treatment Scale | Service user | - Intent to Attend and Complete Treatment Scale (28) | Every 6 months (including Baseline) |
| Symptoms | Service user | - Modified Colorado Symptom Index (MCSI) (27) | Every 6 months (including Baseline) |
| Recovery | Service user | - Questionnaire about the Process of Recovery (QPR) (38) | Every 6 months (including Baseline) |
| Life Outlook | Service user | - A question was derived from suggested questions from Nev Jones (personal communication, August 2020) to capture role satisfaction  - Question 1 from Personal Wellbeing Index(39)  - Construct prioritized in outcomes focus groups | Every 6 months (including Baseline) |
| Hospitalizations | Service user | - EPINET CAB | Every 6 months (including Baseline) |
| Traumatic Events and Experiences | Service user | -Life Events Checklist (LEC-5) (40) and PTSD Checklist for DSM-5 (PCL-5) (41) | Every 6 months (including Baseline) |
| Traumatic Events and Experiences | Service user | - Child and Adolescent Trauma Screen (CATS) – Youth Report (Age 7-17) (42) | Every 6 months (including Baseline) |
| Shared Decision Making and Treatment Satisfaction | Service user | -Shared Decision Making Questionnaire (SDM-Q-9) (43)  - Kickstart Satisfaction: domain required for primary aims | Every 6 months (including Baseline) |
| Pathways to Care | Clinician | - EPINET CAB(26) | Enrollment |
| Diagnoses and Duration of Untreated Psychosis (DUP) | Clinician | - EPI-CAL modified this survey from EPINET CAB(26) to include more specific and exhaustive list of DSM-5 diagnoses | Every 6 months (including Baseline) |
| Family and/or Support Person Involvement | Clinician | - EPINET CAB(26) | Every 6 months (including Baseline) |
| Risk to Self/Others | Clinician | - EPI-CAL researchers modified from EPINET CAB(26) | Every 6 months (including Baseline) |
| Health | Clinician | - EPI-CAL researchers modified from EPINET CAB(26) | Every 6 months (including Baseline) |
| Medications | Clinician | - EPINET CAB(26) | Every 6 months (including Baseline) |
| Service Use | Clinician | - EPI-CAL researchers | Every 6 months (including Baseline) |
| Functioning | Clinician | - Either Global Functioning: Role(44) and Global Functioning: Social (45) or MIRECC GAF(46) | Every 6 months (including Baseline) |
| Symptoms | Clinician | One of:  - Brief Psychiatric Rating Scale (BPRS) (47)  - Positive and Negative Symptoms of Schizophrenia Scale (PANSS-6) (48)  - COMPASS-10 (49) | Every 6 months (including Baseline) |
| Demographics and Background of Primary Support Person (PSP) | PSP | - A question included to measure exposure to poverty at a young age, which was indicated as a risk factor for persistent poverty(29).  - A question derived from ABCD Study(50) (<https://abcdstudy.org>) and Deanna Barch (Personal Communication, September 2020)  - Collateral report for the service user-self report question. Response options were informed from literature(35) and stakeholder feedback during focus groups. | Enrollment |
| Demographics and Background of Primary Support Person | PSP | - EPI-CAL Researchers | Every 6 months (including Baseline) |
| Legal Interactions and Related | PSP | - Collateral report for the service user-self report question. Response options were informed from literature(35) and stakeholder feedback during focus groups. | Every 6 months (including Baseline) |
| Family Impact | PSP | - Burden Assessment Scale (51) | Every 6 months (including Baseline) |
| Symptoms | PSP | - Modified by EPI-CAL team for collateral report from original MCSI (27) | Every 6 months (including Baseline) |
| Medications | PSP | - Modified by EPI-CAL team for collateral report from original GASS (36) | Every 6 months (including Baseline) |

**^a^** For measures without a single validated source, our team and other collaborators created the questions based on multiple sources.

[1.    Tryon VL, Nye KE, Savill M, Loewy R, Miles MJ, Tully LM, Padovani AJ, Tancredi DJ, Melnikow J, Ereshefsky S, Sharma N, McNamara AP, Kado-Walton M, Hakusui CK, Miller C, Nguyen KLH, Safdar M, Padilla VE, Smith L, Wilcox AB, Banks LM, Hayes SL, Pierce KM, Muro K, Shapiro DI, Bolden-Thompson KA, Botello RM, Grattan RE, Zhang Y, Hotz B, Dixon L, Carter CS, Niendam TA. The California collaborative network to promote data driven care and improve outcomes in early psychosis (EPI-CAL) project: rationale, background, design and methodology. BMC Psychiatry 2024 Nov 14;24(1):800. PMID:39543502](https://sciwheel.com/work/bibliography/17212680)
